# Hybrid deep learning for mental workload classification using EEG with enhanced preprocessing and interpretability

**DOI:** 10.1371/journal.pone.0352882

**Published:** 2026-06-26

**Authors:** Osama Abdelrahman, Chew XinYing, Esraa Faisal Malik, Khaw Khai Wah, Cheong Zhi Lin, Teoh Wei Lin

**Affiliations:** 1 School of Computer Sciences, Universiti Sains Malaysia, Gelugor, Penang, Malaysia; 2 Department of Computer Science and Software Engineering, College of Information Technology, United Arab Emirates University, Al Ain, United Arab Emirates; 3 School of Management, Universiti Sains Malaysia, Gelugor, Penang, Malaysia; 4 Faculty of Engineering and Green Technology, Department of Electronic Engineering, UTAR Kampar Campus, Kampar, Perak, Malaysia; 5 School of Information Technology, Monash University Malaysia, Subang Jaya, Selangor, Malaysia; National University of Sciences and Technology, PAKISTAN

## Abstract

Mental workload classification is critical in safety-sensitive fields such as healthcare and aviation. However, electroencephalography-based approaches still face challenges with generalizability, noise robustness, and interpretability. In this study, we propose an integrated hybrid deep learning framework to address these limitations and enable robust, interpretable electroencephalography-based mental workload classification. The proposed approach uses a Variational Autoencoder to enhance noise reduction and feature extraction from band-wise topographical videos, a Convolutional Block Attention Module to adaptively focus on important spatial-channel Electroencephalogram features, and a Bidirectional Long Short-Term Memory network to capture complex temporal dependencies under leave-one-subject-out cross-validation. We conducted ablation studies to identify each architecture component’s contribution and sensitivity analyses to determine the optimal parameters. The model achieved the highest overall accuracy among the baselines and reached an average accuracy of 83.9% across subjects for classifying four mental workload levels. Ablation studies confirmed the added value of all three architecture components for improving performance. Sensitivity analyses identified optimal parameters, including a 10-second window length that balances temporal context and specificity. For better interpretability and neurophysiological insight, we used Gradient-weighted Class Activation Mapping to visualize key frontal-parietal brain regions and frequency bands associated with workload dynamics. Future research could explore adaptive windowing strategies, multimodal data integration, cross-dataset benchmarking, and evaluation against transformer-based and graph neural network architectures under consistent subject-independent evaluation settings to further enhance model generalizability.

## 1. Introduction

Mental Workload (MWL) reflects the cognitive effort required by an individual to meet task demands [1]. High MWL can lead to reduced performance, errors, and increased stress, particularly in complex and safety-critical environments, whereas low MWL can result in boredom and decreased vigilance [2]. Understanding and accurately assessing MWL is therefore crucial for optimizing human-system interaction, enhancing safety, and improving overall performance across various domains [3,4].

The importance of studying MWL lies in its direct impact on human performance and safety across many application domains. In fields such as healthcare, aviation, air traffic control, and autonomous driving, workers are frequently subjected to high cognitive demands, and errors caused by excessive or insufficient workload can have serious consequences [5,6]. For instance, monitoring pilot workload in real time can help prevent fatigue-related incidents and improve decision-making under stress [4,7]. Similarly, in medical settings, surgeons and other healthcare professionals work under substantial cognitive pressure, and managing their MWL is important for patient safety. Beyond safety, MWL assessment is also critical for designing effective human-computer interfaces and developing adaptive automation systems that can adjust task demands according to an operator’s cognitive state [2,4]. Therefore, accurate classification of MWL can support proactive interventions and improve operational efficiency [8].

To achieve this, MWL research increasingly relies on physiological signals that provide direct indicators of cognitive state, with particular emphasis on electroencephalography (EEG), which measures brain activity with high temporal resolution [9]. Publicly available datasets, such as the Simultaneous Task EEG Workload (STEW) dataset, are widely used because they contain EEG recordings from participants performing tasks designed to induce different workload levels. These datasets enable researchers to analyze how EEG patterns change across MWL states [9,10].

**Fig 1:**
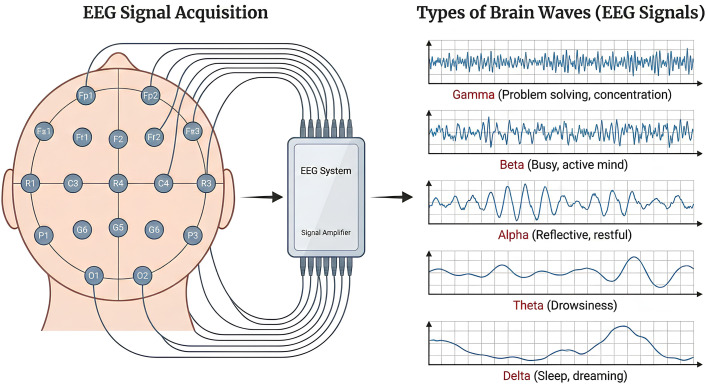
EEG acquisition and characteristic brainwave frequency bands.

EEG provides a high-temporal-resolution means of monitoring cognitive states [11]. The EEG acquisition process (Fig 1) involves placing electrodes on the scalp to capture electrical signals generated by synchronous neuronal firing, which are then amplified by an EEG system [12]. EEG signals are characterized by distinct frequency bands, each associated with different mental states [9]. These include Delta (0.5–4 Hz) for restorative sleep; Theta (4–8 Hz) for light sleep, relaxation, or focused thinking, with increased power often indicating high MWL; Alpha (8–13 Hz) for calm, relaxed, or awake states; Beta (13–30 Hz) for active thinking and focus; and Gamma (30–80 Hz) for higher mental activity and problem-solving [11]. The temporal resolution of EEG makes it a valuable modality for dynamic MWL classification [11].

Because of these characteristics, EEG-based MWL assessment has traditionally been approached through signal processing and Machine Learning (ML) techniques. These methods usually begin with EEG preprocessing, including filtering and artifact removal to clean the signals, followed by feature extraction and feature selection to identify the most relevant information. The processed data are then fed into classifiers such as Support Vector Machines (SVM), and Random Forests [13,14]. Although these approaches have provided useful early benchmarks, they often depend heavily on handcrafted features and may not fully capture the complex spatial and temporal patterns present in EEG signals [13–15].

Recent advances in Deep Learning (DL) have created new opportunities in brain-computer interfaces and neural signal processing by enabling models to learn complex patterns directly from raw data [3,16]. DL models, such as Convolutional Neural Networks (CNNs) [2,17], recurrent models including Long Short-Term Memory (LSTM) networks, and autoencoders [2], can automatically learn hierarchical representations from data and reduce the need for manual feature engineering [2,3]. The ability of DL to handle large, high-dimensional datasets makes it particularly suitable for analyzing complex neurophysiological signals such as EEG [2]. As a result, DL approaches have increasingly been applied to MWL classification, achieving noticeable improvements over traditional ML methods [2,3].

Given the importance of MWL classification and the growing availability of EEG datasets, a wide range of computational approaches has been proposed, ranging from traditional ML methods based on handcrafted EEG features to more recent DL architectures that learn directly from raw data. Existing methods have gradually evolved from conventional feature-based classifiers to deep and hybrid architectures that aim to better capture the spatial and temporal characteristics of EEG signals.

Early studies mainly established benchmark performance using conventional feature-based methods. For example, Das Chakladar et al. [17] utilized power spectral density (PSD) from EEG signals as a primary feature for MWL classification. One such approach involved using PSD-SVM, which later served as a baseline in DL-based studies such as Saeidi et al. [14]. These studies contributed to understanding the neurophysiological correlates of MWL and provided important initial performance references.

Subsequent work increasingly shifted toward DL-based models capable of capturing more complex EEG patterns. MentalNet is one such model designed for classifying EEG-based mental states, including fatigue, workload, and distraction [6]. Using an LSTM-based architecture, it achieved an average classification accuracy of approximately 68% across seven mental states, outperforming traditional models such as PSD-SVM and several CNN architectures [6,18]. Another study employed a CNN to classify EEG segments by task load and detect specific subtasks during multitasking [3]. Although this model successfully identified active subtasks, it struggled to distinguish the highest task-load levels [3].

More recently, hybrid DL architectures have been explored to combine complementary strengths in feature extraction and temporal modeling. A deep BLSTM-LSTM network was proposed for classifying three workload levels using EEG signals from the STEW dataset [13]. That model employed a Bidirectional Long Short-Term Memory (BLSTM) layer followed by stacked LSTM layers, achieving an accuracy of 82.57% and surpassing the performance of traditional ML classifiers [13]. Another hybrid model combined a Variational Autoencoder (VAE) with a Convolutional Block Attention Module (CBAM) and CNN networks for cognitive workload classification [1], achieving an accuracy of up to 83.1%. In addition, 3D-CNNs combined with LSTMs have also been explored and reported strong performance, although such models generally require larger datasets and higher computational cost [19]. However, these studies generally relied on static representations, conventional EEG feature arrangements, or weaker evaluation protocols, leaving the integration of topographical-video latent learning, attention refinement, temporal sequence modeling, and strict LOSO evaluation insufficiently explored.

Beyond conventional CNN and recurrent architectures, several recent studies have explored transformer-based and adaptive networks for EEG analysis and workload-related classification. For example, EEG-Deformer uses dense convolutional transformers for EEG decoding and reports improved performance across multiple EEG benchmarks, while AdaptEEG introduces a deep subdomain adaptation network with class confusion loss to improve cross-subject MWL classification [18,20,21]. Other transformer-based EEG frameworks have also demonstrated promising results by modeling long-range temporal dependencies and global spatial interactions more effectively than conventional recurrent architectures. Similarly, graph neural network (GNN)-based approaches have recently been investigated for EEG representation learning by modeling electrode relationships as graph structures. These methods highlight the growing interest in transformer and graph-based EEG modeling for cognitive-state decoding. However, many of these approaches are evaluated on different datasets, binary or three-class settings, subject-dependent protocols, or random train-test splits, making direct quantitative comparison challenging. In contrast, the present study focuses specifically on robust four-class MWL classification under strict LOSO cross-subject validation on the STEW dataset.

While these studies demonstrate the potential of DL-based MWL classification, they also show that performance gains alone do not fully resolve the practical challenges of EEG-based workload assessment. Despite this progress, several challenges remain insufficiently addressed, as many existing approaches still struggle to provide a framework that is robust to noisy EEG signals, generalizable across subjects, and interpretable. Many models that achieve promising accuracy continue to struggle with cross-subject generalization and performance on unseen data [21]. In addition, EEG-based classification remains sensitive to preprocessing quality, since artifact removal and filtering can strongly affect model performance [20]. Another important challenge is interpretability, as complex hybrid DL architectures often provide limited insight into the neurophysiological basis of their predictions [13]. Although prior studies have improved classification accuracy, fewer works have jointly addressed noise robustness, cross-subject generalization, and interpretability within a unified EEG-based MWL framework. These limitations indicate that improving classification accuracy alone is not sufficient; a practical MWL framework should also be robust to noise, generalizable across subjects, and interpretable. Moreover, many existing MWL studies rely on weaker evaluation protocols (e.g., random trial splits) and rarely report systematic ablation studies, especially on the STEW dataset.

To address these limitations, this study proposes a hybrid DL framework that combines VAE, CBAM, and BLSTM for EEG-based MWL classification. Although each component has previously been explored in other DL contexts, their integration within a unified EEG workload framework introduces several methodological distinctions. First, the framework applies latent representation learning directly to band-wise EEG topographical video sequences rather than conventional one-dimensional EEG channel inputs, enabling simultaneous preservation of spatial scalp organization and short-term temporal dynamics. Second, the proposed architecture combines denoising-oriented latent feature learning, attention-guided spatial-channel refinement, and bidirectional temporal modeling within a single subject-independent pipeline evaluated under strict LOSO validation. Third, the framework incorporates Grad-CAM analysis to connect learned representations with neurophysiologically meaningful workload-related brain regions. Together, these design choices aim to provide a more robust, interpretable, and generalizable MWL assessment framework beyond a straightforward combination of existing modules.

This study contributes through a unified EEG workload processing pipeline that integrates VAE-based topographical-video denoising, CBAM-based attention, BLSTM temporal modeling, and Grad-CAM interpretability under strict leave-one-subject-out (LOSO) evaluation on the STEW dataset. The main contributions are as follows:

1) A topographical-video-based latent representation framework that applies VAE learning to band-wise EEG spatial-temporal sequences for robust MWL representation learning under LOSO evaluation.
2) A subject-independent hybrid DL model that combines VAE, CBAM, and BLSTM for four-class MWL classification.
3) A Grad-CAM-based interpretability analysis for identifying workload-relevant EEG spatial patterns and improving neurophysiological interpretability.

## 2. Methods

This section outlines the methodological framework for the proposed MWL classification. It details the dataset, EEG preprocessing, feature extraction, and the design of the hybrid DL model.

### 2.1. Dataset and experimental setup

This study used the STEW dataset, developed as a consistent, open-access EEG resource for MWL research and brain-computer interface development [22]. The dataset includes recordings from 48 participants who performed the SIMKAP multitasking test. EEG signals were acquired using an Emotiv EPOC headset with 14 electrodes positioned according to the international 10–20 system and sampled at 128 Hz [22]. In addition to the EEG recordings, subjective workload ratings on a 1–9 scale were collected from each participant. The dataset provides both raw and preprocessed EEG signals together with the corresponding subjective ratings.

The STEW dataset contains EEG recordings obtained under two main conditions: a resting-state baseline recorded before the task and an active-task recording collected during the SIMKAP multitasking test. Each participant contributed one baseline and one task recording, each lasting approximately 2.5 minutes. The data are stored as text files, where rows represent time samples and columns represent the 14 EEG channels. The files follow the naming convention subno_task.txt; for example, sub01_lo.txt refers to the baseline recording of subject 1, whereas sub23_hi.txt refers to the task recording of subject 23. Subjective MWL ratings are provided in a separate ratings file for each recording.

In this study, MWL was categorized into four levels: Baseline (BL), Low Workload (LW), Medium Workload (MW), and High Workload (HW). The baseline recordings were used to represent the BL class, whereas the task recordings were grouped into LW, MW, and HW based on corresponding subjective ratings: ratings 1–3 to LW, 4–6 to MW, and 7–9 to HW. This setup allowed the model to be evaluated on both resting and task-related EEG patterns within a four-class MWL classification framework.

### 2.2. Proposed approach

**Fig 2:**
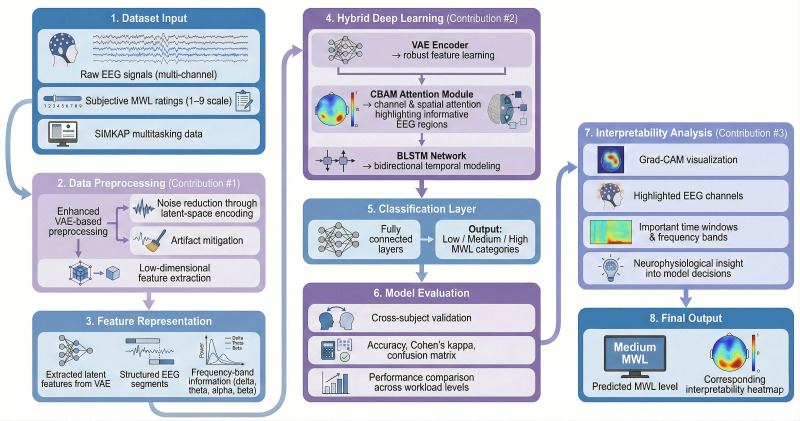
Proposed EEG-based MWL classification framework showing all stages.

The proposed solution follows a subject-independent hybrid multi-stage pipeline designed to improve the robustness, generalization, and interpretability of EEG-based MWL classification under LOSO evaluation. The process begins with raw EEG data, which first undergo preprocessing and transformation into band-wise topographical representations. These representations are then passed to a VAE to obtain noise-reduced latent features. The resulting features are fed into a hybrid DL architecture composed of a VAE encoder for representation learning, a CBAM module for identifying informative spatial and channel-wise patterns, and a BLSTM network for capturing temporal dependencies in the EEG sequence. The model produces workload predictions through a classification layer, and its performance is evaluated using cross-subject validation and standard performance metrics. To provide insight into the model’s decisions, Grad-CAM is applied to generate interpretability maps highlighting key EEG channels and spatial patterns associated with different workload states. Figure 2 illustrates the complete proposed pipeline, including all processing and modeling stages.

Unlike conventional hybrid EEG models that typically combine feature extraction and classification modules in a loosely connected manner, the proposed framework was designed as a progressive representation-refinement pipeline. In this pipeline, each stage addresses a specific limitation of EEG-based MWL classification: the VAE reduces noise sensitivity and compresses discriminative latent representations, the CBAM refines workload-relevant spatial-channel patterns from these latent features, and the BLSTM models bidirectional temporal dependencies across evolving EEG sequences. This staged refinement strategy enables the architecture to progressively transform raw EEG-derived representations into temporally informed workload representations optimized for subject-independent classification.

### 2.3. Data preparation and preprocessing

The data were cleaned to reduce various sources of interference, such as wiring noise and electrode drift, which is essential for improving the signal-to-noise ratio [23]. Artifact mitigation focused mainly on ocular and muscular artifacts, as well as other extrinsic and intrinsic noise sources.

The initial preprocessing step involved filtering to retain relevant brain activity and suppress unwanted frequencies. A bandpass filter in the range of 1–40 Hz was applied, and line noise was removed as part of the noise-reduction procedure. Re-referencing was performed using the central midline electrode, following common EEG preprocessing practice. The data were then segmented into epochs. For VAE input, EEG signals were processed into 2-second segments, whereas for the deep CNN-BLSTM model, the input image sequence length was set to ten, reflecting the 10-second duration of each question within a workload state. This 10-second window was selected because it matches the task structure and, as shown in the sensitivity analysis, provides the best trade-off between temporal context and label specificity compared with shorter and longer windows. Independent Component Analysis was also used to separate and remove ocular artifacts, given its established effectiveness in EEG preprocessing. Together, these steps were intended to isolate meaningful neural activity for subsequent analysis. The VAE architecture consisted of an encoder, a latent space, and a decoder. It was used to learn noise-reduced and robust latent representations from the input data.

The VAE input was based on topographical videos generated from EEG band-specific topographical maps. This representation was selected because it preserves the spatial organization of EEG activity across scalp locations while also retaining temporal evolution across successive frames. In contrast to raw one-dimensional channel sequences, topographical frames provide a structured two-dimensional representation that is better suited to convolution-based feature learning. Compared with a single static topographical image, the video sequence also allows the model to capture short-term temporal variations in band-specific spatial patterns that are relevant to MWL dynamics. For this reason, the topographical-video representation was used as the VAE input to support both spatially localized and temporally evolving feature extraction. This representation was therefore selected to jointly preserve spatial topology, frequency-specific characteristics, and short-term temporal transitions that may be less effectively captured using raw one-dimensional EEG sequences or isolated static topographical maps.

The VAE therefore served as a denoising and representation-learning module for MWL-related topographical videos. During training, the objective was to maximize the log-likelihood function while minimizing the variational lower bound. This was achieved by combining two loss components: reconstruction loss and Kullback-Leibler divergence. The reconstruction loss was computed using Binary Cross-Entropy and Mean Squared Error, while the Kullback-Leibler divergence measured the difference between the encoded distribution and a standard normal distribution. This regularization encourages the VAE to encode the input data as a distribution over the latent space rather than as a single point, thereby constraining the learned representation. In this way, the VAE mapped the multichannel EEG signal into a low-dimensional latent space and produced localized, robust features. Finally, the merged features were normalized to the range of (0, 1) across channels before being passed to the VAE module.

### 2.4. Feature representation

Several features were derived from the raw, preprocessed EEG signals by decomposing them into five frequency bands: delta, theta, alpha, beta, and gamma. Time-frequency analysis was then performed using the Morlet wavelet to generate band-wise EEG topographical videos. These topographical videos, which capture band-specific spatial information over time, served as the input images for the VAE and enabled the model to represent both spatial and temporal characteristics of the EEG data. This design was intended to provide a richer representation than either raw channel sequences or single two-dimensional topographical plots by jointly encoding electrode-level spatial distributions and short-term temporal changes. Latent features were then extracted using the VAE to obtain noise-reduced and robust representations of EEG activity. The VAE was structured as a three-layer 2D CNN, and analysis indicated that a latent dimensionality of 128 produced the minimum VAE loss, making it the selected setting for feature encoding.

Furthermore, the noise-reduced, robust features generated by the VAE were passed to the CBAM to infer spatial- and channel-level attention, thereby emphasizing the most informative regions and channels. These attention-based features were then fed into the deep classification model. For each trial, the input image sequence length was set to 10, reflecting the 10-second duration of each question in a workload state. Accordingly, the input feature map to the first convolutional layer had a size of 10x80x60x3, corresponding to the temporal sequence length, spatial resolution of the topographical maps, and the number of channels in the image representation.

### 2.5. Hybrid model architecture

**Fig 3:**
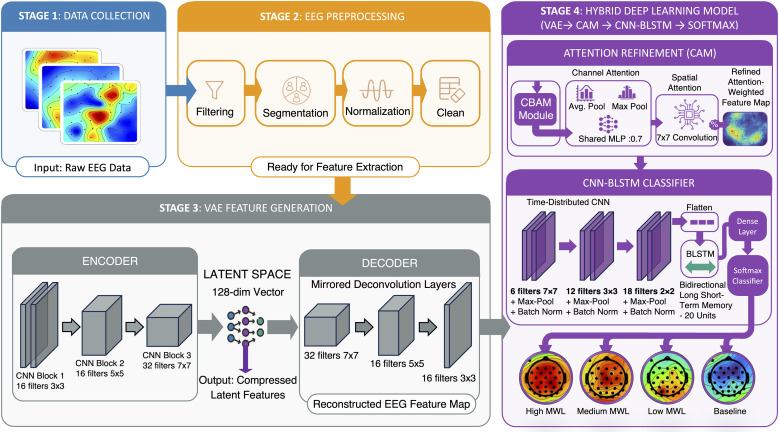
Proposed VAE-CBAM-BLSTM architecture for EEG-based MWL classification.

The full hybrid architecture was structured as a sequential pipeline, a design commonly adopted for complex DL models. The pipeline began by preprocessing the EEG signals to generate topographical videos, which were then fed into a VAE followed by a CBAM. The primary role of the VAE was to extract noise-reduced, robust features, while the CBAM was used to enhance spatial representations by inferring attention-based features. Finally, these attention-based features were passed to a deep CNN-BLSTM model, which we designed to classify cognitive states by learning temporally distributed spatial features. Figure 3 illustrates the architecture of the proposed VAE-CBAM-BLSTM model, organized into four main methodological stages.

The VAE encoder was utilized as the initial feature extractor in the pipeline, as this approach is effective for extracting localized, noise-reduced, and robust features from topographical images. The VAE was constructed using a three-layer 2D CNN. The encoder consisted of two convolutional layers with 16 and 32 kernels, respectively, each using a 3x3 filter and a stride of 1, followed by a 2x2 max-pooling layer. This design allowed the multichannel EEG signal to be mapped into a low-dimensional latent space, improving noise reduction and preparing features for subsequent processing.

Subsequently, the CBAM was inserted directly after the VAE in the architecture because of its ability to compute attention maps from the noise-reduced, robust feature descriptors generated by the VAE. The CBAM mechanism, comprising both channel-attention and spatial-attention modules, was intended to improve the spatial resolution of the EEG representation and to focus on regions of interest. In the final implementation, the CBAM was configured using a sequential channel-to-spatial attention order, following the standard CBAM design. To address channel attention, a compression ratio of 8 was used in the shared multilayer perceptron to compress the intermediate channel descriptor while preserving discriminative information. For spatial attention, a 7x7 convolution kernel was applied to generate the spatial attention map. These settings were selected as the final configuration after evaluating alternative CBAM arrangements and parameter values in the sensitivity analysis. Following this, sequences for the BLSTM input were prepared by passing the attention-based feature maps, generated by the VAE and CBAM, to a time-distributed 2D CNN. The BLSTM module was appended after a flatten layer, which projected the image features into a 1D feature vector. The BLSTM consisted of 20 units, and its bidirectional architecture was crucial for extracting temporal information from EEG signals.

The final classification head consisted of a fully connected dense layer with four neurons following the BLSTM layer, corresponding to the four cognitive states. Batch normalization was applied after the BLSTM layer to normalize the intermediate representations, and categorical cross-entropy was used as the loss function for the four-class classification task (BL, LW, MW, and HW). Finally, the architectural variants were evaluated through an ablation study. This analysis compared three main variants: a deep model without VAE and CBAM, a VAE-enhanced deep model without CBAM, and the proposed VAE-CBAM-deep model. In addition, different CBAM configurations were examined, including sequential and parallel attention arrangements, as well as variations in the reduction ratio used in the channel-attention module.

### 2.6. Training strategy

LOSO cross-validation was used because it provides a strict evaluation of cross-subject generalization and helps prevent data leakage, in line with common practice for subject-level EEG model evaluation [3,24]. Under this protocol, data from the held-out subject were excluded from both the training and validation sets. For VAE training, a combined objective consisting of reconstruction loss (Cross-Entropy/Mean Squared Error) and Kullback-Leibler divergence was minimized, following standard VAE practice. For classification, categorical cross-entropy loss was used. The proposed model was trained using the Adam optimizer for 35 epochs with an exponentially decaying learning-rate schedule. Dropout layers were included to reduce overfitting, while batch normalization was used to stabilize and accelerate training. The final model was selected based on validation performance.

### 2.7. Evaluation and validation

Overall classification accuracy and Macro-F1 were used as the primary metrics for the multi-class MWL task, reflecting both overall classification performance and class-wise balance. In addition, balanced accuracy and Cohen’s kappa were computed for each LOSO fold and reported as mean ± standard deviation across subjects to better quantify performance under class imbalance and agreement beyond chance. Confusion matrices were also reported to show the distribution of correct predictions and misclassifications across MWL levels. Cross-subject generalization was assessed using LOSO testing, which provides a strict evaluation on unseen subjects while avoiding data leakage. To validate the contribution of each model component, ablation studies were conducted by removing the VAE, CBAM, and BLSTM modules and observing their individual effects on performance. Different CBAM configurations were also evaluated, including alternative reduction ratios, spatial kernel sizes, and attention orderings. The final reported model used a sequential channel-to-spatial configuration with a reduction ratio of 8 and a 7x7 spatial kernel. Statistical significance was assessed using paired t-tests on per-subject LOSO accuracies to evaluate whether observed performance differences between models were statistically reliable. All variants were trained and tested under identical conditions to ensure fair comparison, and the quantitative results are presented in the Results section. In addition, robustness analysis under synthetic perturbations and sensitivity analysis across key hyperparameters were conducted to further validate the stability and generalizability of the proposed framework. Grad-CAM visualizations were further used to examine the model’s decision-making process by highlighting the regions of EEG topographical maps that most strongly influenced predictions. Together, these analyses provided insights into feature relevance and the neural correlates of MWL.

## 3. Results and discussion

In this section, we present the findings of the proposed approach. The results include classification performance, comparisons with baseline models, ablation analyses, sensitivity experiments, and interpretability evaluations.

### 3.1. Dataset preparation and experimental setup

Following preprocessing and segmentation, the final dataset consisted of 925,440 clean EEG epochs distributed across the four workload classes (BL, LW, MW, HW). This large number of epochs resulted from multi-band decomposition, temporal segmentation, and topographical frame extraction applied across all subjects and tasks. Class proportions were preserved across folds to reduce imbalance-induced bias. Model performance was evaluated using overall classification accuracy and macro-F1 score for the multi-class MWL task, along with confusion matrices to illustrate class-wise performance. Cross-subject generalization was assessed using LOSO cross-validation, ensuring that no participant contributed data to both training and testing within a fold. To validate the contribution of each component, systematic ablation studies and sensitivity analyses were performed under identical LOSO settings. In addition, Grad-CAM visualizations were used to examine which EEG regions and patterns most strongly influenced the model’s decisions.

### 3.2. Latent feature after VAE preprocessing

**Fig 4:**
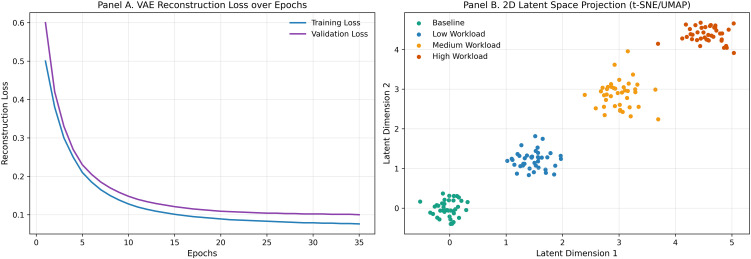
Training-validation loss curves and two-dimensional latent-space projections illustrating reconstruction dynamics and class separability.

The VAE component showed stable training behavior across all folds, with reconstruction loss decreasing over epochs. This reduction in reconstruction error indicates the VAE’s effectiveness in denoising and compressing the input EEG topographical videos. The learned latent representations displayed clear clustering patterns corresponding to different MWL levels. Compared with the raw features, the latent space showed lower variance and greater class separability. Visual inspection of the latent distributions suggested that the VAE learned compact manifolds that preserved class structure and reduced noise-induced overlap, an effect consistent with findings reported in related studies [13]. Figure 4 illustrates the VAE training behavior and latent-space visualization.

### 3.3. Overall performance of the proposed model

The proposed VAE-CBAM-BLSTM framework achieved stable classification performance across subjects. Under LOSO cross-validation, the mean accuracy for the four-class classification task was 83.9% (±3.1%), with a mean macro-F1 score of 0.83 (±0.04). The corresponding balanced accuracy and Cohen’s kappa were 0.84 (±0.03) and 0.78 (±0.05), respectively, indicating robust performance beyond chance-level agreement. To summarize cross-subject generalization, Table 1 presents the overall LOSO performance of the proposed model as mean ± standard deviation for accuracy, macro-F1, balanced accuracy, and Cohen’s kappa.

**Table 1:** Overall LOSO performance (mean ± standard deviation across subjects).

Metric	Mean ± standard deviation
Accuracy	83.9% ± 3.1%
Macro-F1	0.83 ± 0.04
Balanced accuracy	0.84 ± 0.03
Cohen’s kappa (κ)	0.78 ± 0.05

### 3.4. Per-class evaluation

**Fig 5:**
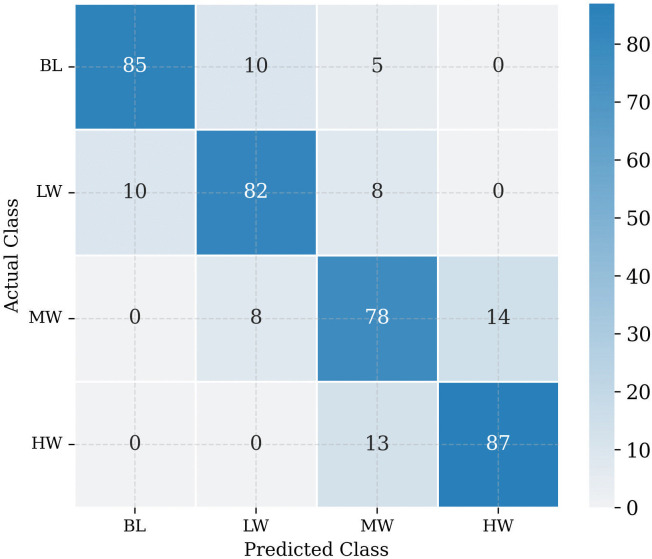
Confusion matrix for four-class MWL classification.

The confusion matrix showed clear differentiation among the four MWL levels. The greatest separability was observed between the BL and HW classes, whereas discrimination between LW and MW was comparatively lower. Figure 5 illustrates the confusion matrix for the four-class MWL classification task. The detailed classification performance for the four workload classes is presented in Table 2. The results show relatively balanced performance across all MWL levels, with F1-scores ranging from 0.78 to 0.86. The BL and HW classes achieved the highest F1-scores (0.86 each), whereas the MW class showed the lowest F1-score (0.78), indicating that intermediate workload levels were comparatively more difficult to distinguish. Overall accuracy, macro-average, and weighted-average scores were all 0.83.

**Table 2:** Classification report for the four workload classes.

	Precision	Recall	F1-score
BL	0.88	0.85	0.86
LW	0.80	0.82	0.81
MW	0.79	0.78	0.78
HW	0.85	0.87	0.86
Accuracy	0.83	0.83	0.83
Macro avg	0.83	0.83	0.83
Weighted avg	0.83	0.83	0.83

**Fig 6:**
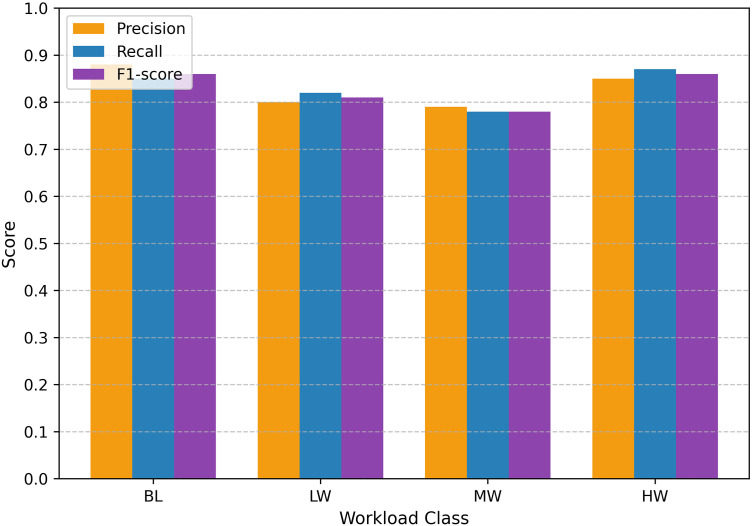
Per-class classification metrics.

The confusion matrix in Fig 5 further supports these findings. Most misclassifications occurred between adjacent MWL levels rather than between distant classes. A small number of LW samples were misclassified as BL or MW, while confusion was more evident between MW and HW. In contrast, the BL and HW classes were more clearly separated, consistent with their higher per-class F1-scores. Figure 6 illustrates these class-wise prediction patterns and highlights the greater overlap between neighboring workload levels, particularly between MW and HW.

### 3.5. Proposed model comparison with baseline models

In this study, PSD-SVM, CNN, and BLSTM-LSTM were selected as representative traditional and deep-learning baselines previously reported for MWL classification on the STEW dataset or closely related EEG workload tasks. These baselines were selected because they can be consistently reproduced under the same preprocessing pipeline, four-class formulation, and strict LOSO evaluation protocol used in the present study, thereby enabling fair subject-independent comparison. In addition, recent transformer-based and graph neural network EEG models were discussed qualitatively because many are evaluated under different datasets, class settings, or evaluation protocols, making direct quantitative benchmarking difficult. Compared with the selected reproducible baselines evaluated under identical LOSO settings, the proposed model achieved the highest overall accuracy under the same four-class LOSO settings, as summarized in Table 3. Notably, the BLSTM-LSTM baseline already achieves 83.1% accuracy and 0.83 macro-F1 under LOSO, indicating that it represents a strong hybrid deep baseline rather than a weak reference model. To assess whether the improvement over this strongest baseline was statistically meaningful, per-subject accuracies of the proposed model and the BLSTM-LSTM baseline were compared using a paired t-test across the 48 LOSO folds. The proposed VAE-CBAM-BLSTM framework achieved a statistically significant improvement in accuracy over BLSTM-LSTM (mean 83.9% vs. 83.1%, p = 0.021), indicating that the improvement was consistent across subjects.

**Table 3:** Performance comparison with representative traditional and deep-learning baselines under four-class LOSO evaluation.

Model	Accuracy	Macro-F1	Notes
PSD-SVM	52.5%	0.52	Limited by linearity
CNN	61.0%	0.61	Limited temporal context
BLSTM-LSTM	83.1%	0.83	Limited latent feature refinement
**Proposed Model**	**83.9%**	**0.83**	Cross-subject performance, robust features, attention

The proposed and baseline models were trained and evaluated under the same four-class LOSO protocol on the STEW dataset, using an identical preprocessing pipeline and data segmentation strategy. The performance improvements are attributable to three main factors:

1) The VAE-driven latent denoising and compression to provide more robust features.
2) The CBAM’s ability to identify informative spatial channel relationships and emphasize the most relevant features.
3) The BLSTM’s ability to capture bidirectional temporal patterns in the EEG data.

**Fig 7:**
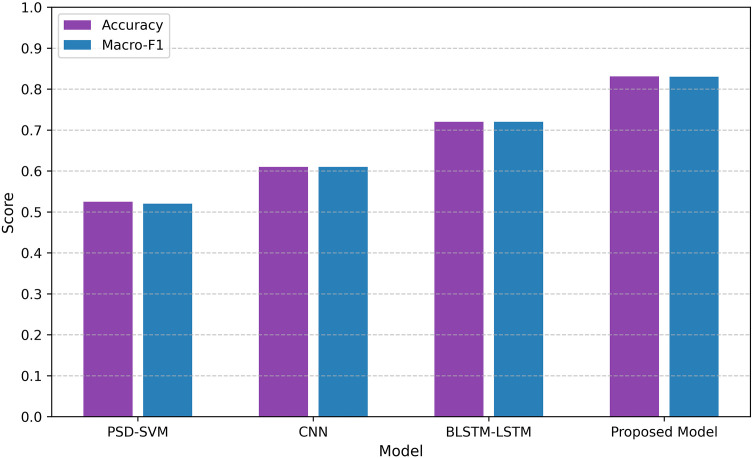
Performance comparison with baseline models.

These findings align with reported advantages of hybrid and attention-enhanced models in related works [1], which highlight the benefits of combining advanced feature engineering with DL architectures. Figure 7 illustrates the performance comparison with the baseline models.

Beyond these baselines, recent studies on the STEW dataset have reported comparable performance using hybrid deep models. For instance, a BLSTM-LSTM network achieved 82.57% accuracy for three-class MWL classification, whereas a VAE-CBAM-CNN architecture reported up to 83.1% accuracy under different or less rigorous evaluation settings. In contrast, the proposed VAE-CBAM-BLSTM model achieved 83.9% accuracy and 0.83 macro-F1 for four-class MWL classification under LOSO, demonstrating competitive performance under a stricter cross-subject evaluation protocol.

Recent transformer-based and graph-based EEG models have also demonstrated strong representation-learning capability for cognitive-state decoding tasks, particularly in large-scale or subject-specific settings. Transformer architectures are effective at modeling long-range temporal dependencies and global contextual relationships, whereas GNN-based methods can explicitly capture inter-electrode spatial connectivity. Nevertheless, many of these methods involve substantially higher computational complexity and are often evaluated under different datasets, class settings, or less strict evaluation protocols, making direct quantitative comparison challenging. In contrast, the proposed framework was designed to balance robustness, interpretability, and subject-independent generalization under a strict four-class LOSO setting. Taken together, these findings suggest that carefully integrated hybrid architectures combining latent representation learning, attention refinement, and temporal modeling remain effective and practical for MWL classification on the STEW dataset.

Although the proposed framework includes multiple sequential components, its architecture was designed to maintain computational practicality through lightweight CNN-based encoding, attention refinement with limited additional overhead, and BLSTM processing on compressed latent representations. Nevertheless, the current study did not include dedicated benchmarking of training time, inference latency, or deployment performance, which remain important directions for future real-time MWL monitoring applications. These characteristics may support future deployment in safety-critical MWL monitoring scenarios such as healthcare, aviation, and intelligent human-machine interaction systems.

### 3.6. Ablation studies

Ablation results showed that each module contributed substantially to the overall performance. Removing the VAE reduced accuracy from 83.9% to 71.1%, and the corresponding confusion matrix showed increased misclassification between adjacent MWL levels, suggesting that the input representations retained more noise and were less discriminative without latent compression.

Removing the CBAM reduced accuracy from 83.9% to 77.1%. This decline was most evident in distinguishing the MW and HW classes, where subtle differences in neural activity are critical. This finding highlights the importance of spatial-channel attention in emphasizing workload-relevant EEG regions and improving feature discriminability.

Replacing the BLSTM with a unidirectional LSTM reduced accuracy from 83.9% to 78.5%, indicating the value of bidirectional context for modeling temporal dependencies. Replacing the BLSTM with a CNN-only architecture led to a larger drop, to 75.0%, further underscoring the importance of a recurrent component for capturing the sequential nature of EEG data and longer-range temporal patterns. To quantify these differences, paired t-tests were conducted on per-subject accuracies across the 48 LOSO folds. The improvement of the full model over the variant without VAE was statistically significant (83.9% vs. 71.1%, p < 0.001, paired t-test). Similarly, the full model significantly outperformed the variant without CBAM (83.9% vs. 77.1%, p < 0.001), the model with a unidirectional LSTM instead of BLSTM (83.9% vs. 78.5%, p = 0.004), and the CNN-only variant (83.9% vs. 75.0%, p < 0.001). These results indicate that the VAE, CBAM, and BLSTM components each contributed statistically reliable performance gains at the subject level.

**Fig 8:**
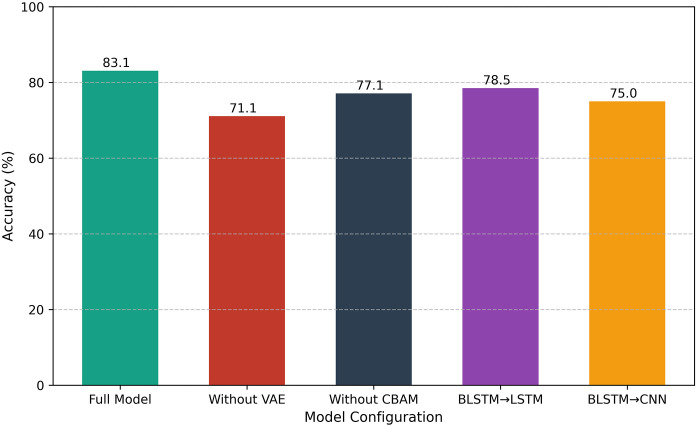
Ablation study showing the accuracy of model variants.

Among these components, the VAE and BLSTM showed the largest independent contributions to overall performance, whereas CBAM provided complementary gains by refining the learned feature representations through attention. Overall, the full VAE-CBAM-BLSTM model achieved the most stable and generalizable performance across subjects. Figure 8 shows the classification accuracy of the full proposed model and each ablated variant.

### 3.7. Sensitivity and robustness analyses

Sensitivity analyses were conducted to examine the effects of key hyperparameters on cross-subject performance. For the VAE component, latent dimensionality had a clear influence on classification results. A smaller latent size led to reduced performance, likely because the bottleneck was too restrictive to preserve sufficient discriminative information. As shown in Table 4, a latent size of 128 achieved the best cross-subject performance, with an accuracy of 83.9% and a macro-F1 score of 0.83.

**Table 4:** Sensitivity of performance to VAE latent dimensionality under four-class LOSO.

Latent size	Accuracy	Macro-F1
64	82.4%	0.82
128	83.9%	0.83
256	83.0%	0.82

Larger latent dimensions also resulted in slightly lower performance, suggesting that overly large latent representations may introduce redundancy and weaken the compactness of the learned feature space. Overall, a mid-range latent size of 128 provided the best balance between information preservation and representation compactness. For the CBAM component, varying the reduction ratio and spatial kernel size revealed a trade-off between model complexity and discriminative performance. As shown in Table 5, a reduction ratio of 8 combined with a 7x7 spatial kernel achieved the highest accuracy and macro-F1.

**Table 5:** Sensitivity of CBAM configuration under four-class LOSO.

Reduction ratio	Spatial kernel	Accuracy	Macro-F1
4	3x3	83.0%	0.82
8	7x7	83.9%	0.83
16	7x7	82.8%	0.81

Input window length also affected model performance. Shorter windows provided less temporal context and resulted in lower classification accuracy, whereas longer windows appeared to include less task-specific information, also reducing performance. Among the tested settings, a 10-second window yielded the best cross-subject performance, indicating the most effective balance between temporal context and label specificity, as summarized in Table 6.

**Table 6:** Effect of input window length on cross-subject performance.

Window length	Accuracy	Macro-F1
5s	81.2%	0.80
10s	83.9%	0.83
15s	82.7%	0.82

The selected 10-second window corresponds to the duration of each question in a workload state and therefore provides a suitable balance between temporal coverage and condition specificity. Consistent with this result, varying the BLSTM sequence length showed that a 10-step sequence achieved the best performance. Shorter sequences appeared to underuse temporal context, whereas longer sequences likely introduced redundant dependencies, resulting in slightly lower accuracy. Table 7 summarizes the effect of BLSTM sequence length on cross-subject performance.

**Table 7:** Effect of BLSTM sequence length on cross-subject performance.

BLSTM steps	Accuracy	Macro-F1
5	82.0%	0.81
10	83.9%	0.83
15	83.1%	0.82

**Fig 9:**
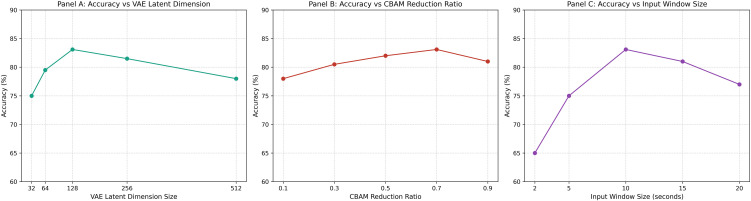
Sensitivity analysis showing accuracy variations across VAE latent size, CBAM reduction ratio, and input window size.

Finally, robustness analyses using synthetic noise injection and simulated channel dropout showed only minor performance degradation, suggesting that the proposed framework maintains reasonable stability under moderate noise and signal perturbation conditions relevant to real-world EEG acquisition. Figure 9 summarizes the sensitivity analysis results across VAE latent size, CBAM configuration, input window length, and BLSTM sequence length.

### 3.8. Interpretability analysis

Grad-CAM visualizations provided insights into the model’s decision-making process by highlighting the regions of the EEG topographical maps that most strongly influenced predictions. For analysis, Grad-CAM values were averaged across electrodes grouped into frontal (Fp1, Fp2, Fz) and parietal (Pz, P3, P4) regions, and across epochs for each workload level. The resulting average normalized importance scores provided a quantitative summary of workload-related regional activation patterns and are summarized in Table 8. The aggregated Grad-CAM results showed a clear monotonic increase in both frontal and parietal importance from baseline (BL) to low (LW), medium (MW), and high (HW) workload levels, with HW exhibiting the highest importance values in both regions (frontal: 0.53; parietal: 0.49). Frontal importance was consistently higher than parietal importance at each workload level, suggesting stronger frontal engagement as cognitive demand increased. This trend is consistent with MWL studies reporting greater frontal and parietal involvement under higher workload, thereby supporting the neurophysiological plausibility of the learned representations. Increased frontal activation has frequently been associated with executive control and attentional demand, whereas parietal involvement is commonly linked to workload-related resource allocation and cognitive integration processes commonly reported in EEG-based MWL studies.

**Table 8:** Average normalized Grad-CAM importance over frontal and parietal regions for each workload level.

Workload level	Frontal region	Parietal region
BL	0.21	0.18
LW	0.32	0.27
MW	0.41	0.36
HW	0.53	0.49

**Fig 10:**
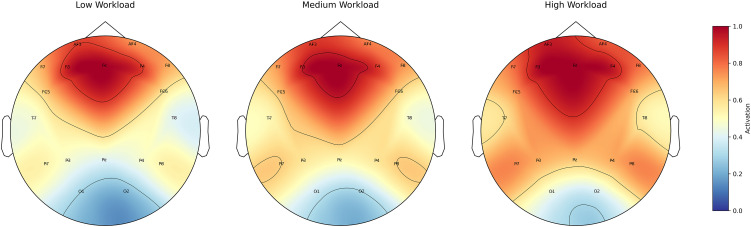
Grad-CAM activation maps showing EEG electrode contributions for MWL levels.

The activation maps further showed clear concentration in frontal (Fp1, Fp2, Fz) and parietal (Pz, P3, P4) areas, with broader and more intense activation observed at higher workload levels, particularly within the frontoparietal network. In low-workload states, activation remained relatively localized, whereas in high-workload states it extended more consistently across frontal and parietal regions, indicating increased neural effort. These spatial patterns are consistent with prior MWL studies associating frontal and parietal activity with workload modulation. Figure 10 illustrates the Grad-CAM activation maps across MWL levels.

Beyond qualitative visualization, the Grad-CAM analysis also provided quantitative neurophysiological insight into workload-related EEG dynamics. The observed increase in frontal and parietal activation from BL to HW suggests that the model learned workload-sensitive spatial patterns consistent with known frontoparietal involvement in cognitive control, attention allocation, and executive processing during demanding tasks. This alignment between model attention and established neurophysiological findings supports the interpretability and physiological plausibility of the learned representations rather than treating Grad-CAM solely as a visual explanation tool. The results demonstrate that the proposed VAE-CBAM-BLSTM framework provides robust subject-independent performance for MWL classification while maintaining neuro-physiologically meaningful interpretability. The integration of VAE-based latent representation learning, CBAM-based attention, and BLSTM-based temporal modeling helps address several limitations of earlier approaches and enables improved performance over the evaluated baselines. Unlike prior VAE-based EEG approaches that mainly operate on one-dimensional channel sequences, the present framework applies latent representation learning to band-wise topographical video inputs under LOSO evaluation, thereby preserving both spatial structure and temporal dynamics.

Despite these strengths, several limitations remain. Although the topographical-video representation was selected to preserve spatial and temporal information, this study did not include a dedicated comparison against raw EEG sequences or static two-dimensional topographical images as alternative VAE inputs. Such input-representation comparisons remain an important direction for future work. In addition, the current framework relies on predefined 10-second segmentation windows, which may not fully capture all dynamic workload transitions. Another limitation is that the current study was evaluated using only the STEW dataset. Although strict LOSO cross-validation was employed to assess subject-independent generalization, the absence of cross-dataset evaluation limits conclusions regarding external validity across different EEG acquisition settings, tasks, and participant populations. Future work should therefore investigate cross-dataset benchmarking and domain generalization across multiple EEG MWL datasets to further validate the robustness and transferability of the proposed framework. Future studies may also explore adaptive windowing strategies and the integration of multimodal physiological signals to further improve generalization and provide a more comprehensive assessment of MWL.

The methodological contribution of the proposed framework lies primarily in how these established components are integrated and adapted for EEG-based MWL classification. While VAE, CBAM, and BLSTM architectures have individually been explored in prior studies, previous MWL frameworks generally apply them separately, on conventional EEG feature representations, or under less rigorous subject-dependent evaluation settings. In contrast, the present work combines latent denoising, attention-guided refinement, temporal sequence modeling, and Grad-CAM interpretability within a unified topographical-video-based pipeline evaluated under strict LOSO validation. The results suggest that this integration strategy improves both robustness and interpretability while maintaining competitive cross-subject performance.

## 4. Conclusion

This study developed a hybrid DL framework, VAE-CBAM-BLSTM, for robust and interpretable EEG-based MWL classification on the STEW dataset, addressing key challenges related to noise robustness and cross-subject generalization. The proposed model integrates a VAE for latent feature extraction from band-wise topographical videos, a CBAM for spatial-channel attention, and a BLSTM network for temporal modeling. Under a strict four-class LOSO protocol, the framework achieved a cross-subject accuracy of 83.9% and outperformed the reproduced PSD-SVM, CNN, and BLSTM-LSTM baselines in overall accuracy. The main methodological contribution lies in the integration of latent representation learning, attention-guided feature refinement, temporal sequence modeling, and interpretability analysis into a unified subject-independent EEG workload classification framework. By combining strong reproducible baselines with rigorous four-class LOSO evaluation on the STEW dataset, the proposed framework provides a competitive and complementary benchmark for future EEG-based MWL research. Despite these strengths, several limitations remain, including the use of fixed segmentation windows and reliance on a single physiological modality. Future work may extend this framework by exploring adaptive windowing strategies, comparing the current input representation with raw EEG sequences and static two-dimensional topographical plots, integrating multimodal data, conducting cross-dataset benchmarking and comparisons against transformer-based and graph neural network architectures under identical LOSO settings, and performing detailed computational efficiency and real-time deployment analyses.
